# The relationship between academic achievement, health behaviors, and school climate among university students

**DOI:** 10.1186/s12889-026-26324-5

**Published:** 2026-01-20

**Authors:** Muhammet Kesin, Halim İşsever

**Affiliations:** 1 Department of Public Health, Safranbolu State Hospital, Karabük, Turkey; 2 Department of Public Health, Faculty of Medicine, Istanbul University, Istanbul, Turkey

**Keywords:** Academic Success, Health Behaviors, Students, Public Health, School Climate

## Abstract

This study aimed to examine whether academic achievement among university students is associated with school climate, health-promoting and protective behaviours, demographic characteristics, accommodation, and employment status. The study population consisted of 1,155 s- and third-year students enrolled at Karabük University. Categorical variables were analyzed using the chi-square test, and relationships between variables were examined with Spearman’s correlation coefficient. Logistic regression analysis was conducted with subjective and objective academic achievement as the dependent variables. Students who were employed, smoked, consumed alcohol, skipped regular breakfast or exercise, or reported irregular sleep patterns were less likely to have higher academic achievement (*p* < 0.05). Students with a history of consulting a doctor for mental health reasons also reported lower academic achievement (*p* < 0.05), whereas those living with their families reported higher achievement (*p* < 0.05). Third-year students had lower academic performance compared to second-year students (*p* < 0.05). School climate and its subdimensions, as well as health-promoting behaviours, were positively associated with both subjective academic achievement and grade point average (*p* < 0.05). These findings suggest that academic achievement may be related to healthy lifestyle habits, a positive school climate, and social support. Universities may consider developing programs that promote healthy living and implementing policies that foster a supportive school environment to help support students’ academic engagement and well-being.

**Supplementary Information:**

The online version contains supplementary material available at 10.1186/s12889-026-26324-5.

## Introduction

Academic achievement is one of the key indicators of an education system and a significant factor in universities' efforts to train students as qualified individuals. Students with low academic achievement generally have lower hopes of entering professional life after graduation compared to those with higher academic success [67]. Education has been associated with economic benefits, such as lower unemployment rates, and social advantages, including lower crime rates. Moreover, it is considered an important tool for disadvantaged groups and may contribute to reducing poverty [37].

Measuring academic achievement solely through exam performance may be insufficient. Environmental factors, personal characteristics, future goals, learning attitudes, and perceptions of success are also important components related to achievement. During the process of successfully completing their education, various factors may be associated with differences in students’ motivation, academic performance, learning progress, and their perspectives on post-graduation life [18]. In this context, many factors are reported to be related to academic achievement. Some potentially supportive factors include support systems (family, friends) and campus resources (libraries, laboratories, extracurricular activities, scholarships). Potentially adverse factors, on the other hand, include language barriers, difficulties in adapting to the education system, learning disabilities, failure in exams, challenges during internships, problems related to online education during the pandemic, socio-cultural differences, daily life hardships, perceived discrimination, interpersonal relationships, and physical and mental health issues [34].

One of these factors, school climate, is defined as students' perceptions of the cultural norms, values, practices, and relationships related to school life [17]. A positive school climate has been shown to buffer the negative effects stemming from poverty and social difficulties and is associated with healthier psychosocial development and well-being [40]. Factors such as the school's physical facilities, teacher–student relationships, the attitudes of school staff, and the safety of the school environment are among the elements that shape perceptions of school climate [6]. School climate is considered a starting point for improving student retention and is positively associated with academic achievement [19]. When school attendance rates decline, students’ academic achievement tends to be lower [23]. Educational institutions with a positive school climate are reported to be associated with higher academic achievement and to foster mutual support, respect, and sincerity. In this context, a positive school climate is regarded as an indicator that the institution has a strong mission, may enhance motivation by supporting student success, and is linked to more favorable educational conditions [58].

Another group of characteristics related to student achievement is health-related behaviors. For individuals to be successful academically, they may need to maintain not only physical health but also mental and psychosocial well-being, in addition to engaging effectively in the learning process [45]. Education has been associated with increased health awareness, while better health has been linked to greater participation in and success at school. Healthy individuals are more likely to complete their education, and educated individuals tend to be more conscious about maintaining their health. This interaction is thought to contribute to both personal well-being and economic productivity [24, 26]. Individuals with lower levels of education exhibit higher rates of chronic diseases and premature mortality [62, 68]. Therefore, promoting students’ health behaviors is viewed as an important factor associated with the efficiency of the educational process and higher academic achievement.

Eliminating or reducing factors that may be associated with poorer academic achievement among university students could help make their educational experience more productive and support a successful start to their professional careers. In the literature, numerous topics have been examined in relation to academic achievement, including coping strategies, emotional intelligence, mental health, achievement motivation, self-efficacy levels, and goal attainment skills. In this study, the term ‘motivation’ is used within the framework of Self-Determination Theory (SDT) to broadly describe students’ academic motivation, including engaging in the learning process or valuing the outcomes of learning [2, 15, 42, 44, 55, 57, 59, 66]. This study is grounded in Bronfenbrenner’s Ecological Systems Theory to contextualize the dynamics between the school environment, health-related behaviors, and student outcomes [13]. Within this theoretical framework, the school climate is conceptualized as a core microsystem in which students engage in direct interactions and proximal processes. According to Bronfenbrenner, proximal processes encompass the activity and behavior patterns in which individuals regularly participate over time. In this regard, the health-related behavior patterns examined in this study were considered as one form of these processes. Furthermore, the quality of the proximal processes occurring within the school environment is associated with levels of “developmental competence,” a construct that includes academic achievement. Therefore, this model provides a theoretical basis for examining school climate, individual processes, and academic outcomes as interrelated components within the same developmental context. However, studies focusing simultaneously on school climate and health-related behaviors remain limited. Building on Bronfenbrenner’s Ecological Systems Theory, this study aims to examine how school climate, health-promoting and protective behaviors, and socio-demographic and environmental factors (gender, age, accommodation type, employment status, smoking, alcohol use, nutrition and sleep patterns) are associated with subjective and objective academic achievement among university students.

## Materials and methods

### Sample size

The study is an analytical cross-sectional research, and its population consisted of all second- and third-year undergraduate students enrolled at Karabük University during the 2023–2024 academic year. First-year students were excluded from the study because they had just started university, and fourth-, fifth-, and sixth-year students were excluded due to undertaking internships outside the city. Assuming an academic achievement rate of 75% among university students and aiming to estimate achievement with an 8% margin of error (using a Type I error of 0.05 and a Type II error of 0.20, power = 0.80), a minimum of 173 participants is required. In this study, a convenience sampling method was employed, and it was aimed to meet the minimum sample size for the identified subgroups. Accordingly, two variables expected to be associated with academic achievement—the type of department attended (health, social, or mathematical sciences) and the class level (second or third year)—were considered in the sample size calculation. The class level consists of two subgroups, and the department type consists of three subgroups. Based on this, considering a minimum of 173 participants per subgroup, it was calculated that a total of 2 (class levels) × 3 (department types) × 173 = 1,038 participants would be required [16].

### Sample distribution

The study data were collected from second- and third-year students within the same departments to minimize the potential confounding influences of factors such as school climate, faculty attitudes, and health behaviors that could vary across departments. For each department type (health, social, and mathematical sciences), it was aimed to reach at least 173 participants in the designated subgroups, and at least one department from each faculty was included in the study. Departments from which data would be collected were selected by lottery; in faculties with insufficient student numbers, multiple departments were selected by lottery, and data collection was completed through one-on-one interviews. Data were collected from 1,300 students in total. Due to incomplete responses to scale questions or language proficiency issues, 145 responses were excluded, resulting in a final sample of 1,155 students Table 1.

**Table 1:** Distribution of students by class level and department type

Department Type^a^	2nd Year	3nd Year	Population	2nd Year Sample	3nd Year Sample	Total
Health	929	896	1825	217	170	387
Mathematics	2293	2100	4393	163	236	399
Social	1666	1094	2760	226	178	369
Total	4888	4090	8978	606	584	1155

^a^The Faculties of Engineering, Architecture, Business, and Economics were classified under the mathematical sciences; the Faculties of Medicine, Dentistry, and Health Sciences under health; and the Faculties of Fine Arts, Communication, Tourism, Islamic Studies, and Literature under social sciences

### Assessment of bias and weighting

Although the targeted subgroup distributions were largely achieved in this study, the desired number of participants in some faculties within the mathematical sciences could not be reached due to high absenteeism rates. Moreover, although the number of students enrolled in mathematical sciences is higher compared to social and health sciences, the amount of data collected from this group remained similar to the other fields. This resulted in the proportional underrepresentation of students in mathematical sciences within the sample, limiting the generalizability of the findings to the entire population. The study was conducted only with students attending classes, and absentee students could not be included. Considering that students who attend classes may have higher academic achievement, this further restricts the generalizability of the research findings.

The incomplete completion of surveys due to language barriers and the exclusion of 145 participants may have impacted the distribution of subgroups. This situation resulted in not reaching the targeted sample size and may have limited the generalizability of the findings. Since the academic term had ended, the missing data could not be completed afterwards. Additionally, probability sampling methods (e.g., stratified sampling, cluster sampling) could not be applied because up-to-date student lists and total enrollment numbers for each department were unavailable. Consequently, the sample could not fully represent the population, further restricting the generalizability of the research findings.

### Instruments used for data collection

The Individual Information Form was employed to assess participants’ socio-demographic characteristics, the Subjective Academic Achievement Scale measured academic achievement, the School Climate Scale for University Students evaluated school climate, and the Health-Promoting and Protective Behaviors Scale identified health-promoting and protective behaviors.

#### Socio-demographic data form

A survey form was developed to collect information on participants’ gender, age, height and weight, department and class level at the university, grade point average, overall health status, smoking and alcohol use, dietary and physical activity habits, history of consulting a physician for mental health issues, employment in income-generating jobs, and type of accommodation. For this study, age, scores on the Health-Promoting and Protective Behaviors Scale, and Grade Point Average (GPA) were categorized based on median values. Individuals at or below the median were classified into one group, while those above the median were classified into another group.

#### Academic achievement

Academic achievement was evaluated as both subjective and objective. Subjective academic achievement was measured using the Subjective Academic Achievement Scale. The Turkish version of the scale was translated by three experts, and the translation was then back-translated into English. Following this process, the Turkish version was reviewed by three experts. The scale was selected for this study to assess subjective academic achievement because it demonstrated a Cronbach’s alpha of 0.82, showed a strong correlation with GPA, and yielded a Cronbach’s alpha of 0.821 in the present study. The Turkish adaptation and psychometric properties of the SAAS were evaluated in a separate sample of 100 participants before data collection for the main study. In addition, the scale demonstrated a significant positive correlation with GPA (r = 0.741, *p* = 0.001). Exploratory factor analysis (EFA) was conducted to assess construct validity; KMO = 0.801, Bartlett’s test *p* < 0.001, and the extracted factor explained 58.65% of the total variance. Objective academic achievement was assessed using data obtained from students’ GPA. The internal consistency of the Subjective Academic Achievement Scale was evaluated using Cronbach’s alpha, indicating a good level of agreement among the items (α = 0.821). All item-total correlations exceeded the critical value of 0.30. Furthermore, Cronbach’s alpha values calculated by removing individual items were either lower than (Table 2 and 3).

**Table 2:** Cronbach’s alpha values of the subjective academic achievement scale

Item	Factor 1 Loading	Item-Total Correlation	Cronbach’s Alpha if Item Deleted
Item 1	0,772	0,623	0,783
Item 2	0,708	0,550	0,805
Item 3	0,791	0,641	0,778
Item 4	0,771	0,628	0,782
Item 5	0,785	0,640	0,782

**Table 3:** Correlation between objective academic achievement and subjective academic achievement

	**GPA Score**	**p**
Subjective Academic Achievement Scale	r: 0,741	0,001

#### Subjective academic achievement scale

The Subjective Academic Achievement Scale (SAAS) was developed by Stadler M. et al. in 2021. The scale consists of 5 items and uses a five-point Likert format. The scale’s Cronbach’s alpha coefficient is 0.82. Scores on the scale range from a minimum of 5 to a maximum of 25. Lower scores indicate lower levels of academic achievement, while higher scores indicate higher levels of academic achievement [56].

#### Health-promoting and protective behaviors scale

The Health-Promoting and Protective Behaviors Scale was developed by Bostan A. N. et al. in 2016. The scale consists of 24 items and uses a five-point Likert format. Its Cronbach’s alpha coefficient is 0.83. Scores on the scale range from a minimum of 24 to a maximum of 120. Lower scores indicate that the individual exhibits fewer health-promoting and protective behaviors [12].

#### School Climate Scale for University Students

School Climate Scale for University Students: The School Climate Scale for University Students was developed by Terzi in 2015. The scale consists of 17 items and uses a five-point Likert format. Its Cronbach’s alpha coefficient is 0.90. Based on item mean scores, values between 1.00 and 2.60 indicate a negative (closed) school climate, 2.61 to 3.40 indicate a moderate level, and 3.41 to 5.00 indicate a positive (open) school climate [60].

### Statistical methods used for data analysis

The collected data were analyzed using SPSS 21.0. Numerical data were presented as mean ± standard deviation or median (min–max), and nominal data as frequency and percentage. Normality was assessed using the Kolmogorov–Smirnov test. Categorical variables were analyzed with the Chi-square test, and relationships between variables were examined using Spearman correlation. The internal consistency of the Subjective Academic Achievement Scale was evaluated with Cronbach’s alpha. In the analysis of subjective academic achievement, the dependent variable was the Subjective Academic Achievement Scale, and independent variables were those with *p* < 0.25; for objective academic achievement, GPA was the dependent variable, and independent variables were those with *p* < 0.25. These relationships were examined using logistic regression (Forward LR). The bootstrap method (1,000 repetitions, 95% confidence interval, BCa correction) was applied to test the robustness of parameter estimates. Statistical significance was considered two-tailed at *p* < 0.05.

Since normality assumptions were not fully met, both GPA and SAAS scores were dichotomized using a median split to allow logistic regression modelling. Median split procedures are widely used in behavioral and health sciences, appearing in approximately 31% of empirical studies [10]. Previous methodological work has demonstrated that median splits can yield valid and robust estimates, particularly when assumptions of normality are violated or when the underlying measurement demonstrates adequate reliability [7, 20]. Iacobucci et al. [30] also reported that median splits perform comparably to continuous analyses in effect direction and classification accuracy. To assess robustness, supplementary linear regression analyses using continuous GPA and SAAS scores were conducted. The direction and significance of the associations remained consistent, supporting the stability of the findings.

## Results

A total of 1,155 students were included in the analyses. The socio-demographic.

characteristics of the participants are presented in Table 4.

**Table 4:** Distribution of socio-demographic characteristics

	**n**	**%**
Gender		
Female	591	%51,1
Male	564	%48,9
Body Mass Index		
Underweight	91	%7,9
Normal	734	%63,5
Overweight	279	%24,2
Obese	51	%4,4
General Health Status		
Good	627	%54,3
Moderate	448	%38,8
Poor	80	% 6,9
Smokers	341	%29,5
Alcohol Users	203	%17,6
Regular Breakfast Consumers	657	%56,9
Regular Exercisers	636	%55,1
Employed in Income-Generating Job	165	%14,3
Healthy Sleep Patterns	512	%44,3
History of Consulting a Physician for Mental Health Issues	196	%17,0
Type of Accommodation		
Dormitory	653	%56,6
With Family	143	%12,4
Rented	359	%31
Class Level		
2nd Year	571	%49,4
3rd Year	584	%50,6
Department Type		
Social	369	%32,0
Mathematical	399	%34,5
Health	387	% 33,5

**Fig. 1:**
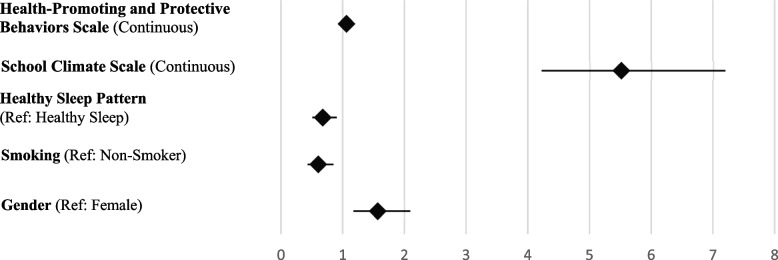
Forest plot of logistic regression analysis results for the subjective academic achievement scale

Variables with p < 0.25 were included in the logistic regression model (Table 5, Fig. 1). The model explained 38.9% of the variance in subjective achievement. Higher school climate scores (OR = 5.512) and health-promoting behaviors (OR = 1.063) increased the odds of higher subjective academic achievement. Students who smoked were 39% less likely, and those with irregular sleep patterns were 31.9% less likely, to report higher achievement. Male students were 57% more likely to report higher subjective achievement than females. Bootstrap validation confirmed model stability.

**Table 5:** Logistic regression analysis results for subjective academic achievement scale

**Logistic Regression Analysis for Subjective Academic Achievement Scale** (0: SAAS ≤ Median, 1: SAAS > Median)							**95% Confidence Interval for OR**	
**Variables**	**B**	**Sh**	**Wald**	**Sd**	**p**	**Exp(B)**	**Lower**	**Upper**
Health-Promoting and Protective Behaviors Scale (Continuous)	0,061	0,011	32,737	1	0,001	1,063	1,041	1,085
School Climate Scale (Continuous)	1,707	0,136	157,467	1	0,001	5,512	4,222	7,195
Healthy Sleep Pattern (Ref: Healthy Sleep)	−0,384	0,145	6,983	1	0,008	0,681	0,512	0,906
Smoking (Ref: Non-Smoker)	−0,495	0,172	8,328	1	0,004	0,610	0,436	0,853
Gender (Ref: Female)	0,451	0,148	9,297	1	0,002	1,570	1,175	2,099

Nagelkerke R^2^ = 0,389; Omnibus Chi-square = 394,222; *p* < 0,001; Hosmer and Lemeshow = *p* > 0,05

**Fig. 2:**
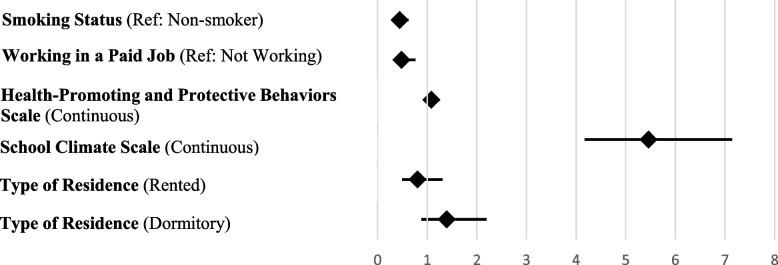
Forest plot showing the results of the logistic regression analysis for GPA

The model explained 44.4% of the variance in GPA (Table 6, Fig. 2). Students who smoked were 56% less likely to have a higher GPA (OR = 0.444). Those working in paid jobs were 52.1% less likely to have a higher GPA (OR = 0.479). School climate (OR = 5.458) and health-promoting behaviors (OR = 1.082) were strongly associated with higher GPA. Accommodation type was significant as a block, although no individual category was statistically significant. Bootstrap results supported the robustness of the estimates.

**Table 6:** Logistic regression analysis results for GPA

**Logistic Regression Analysis for GPA** (0: Median value and below GPA, 1: Above median GPA)							**95% Confidence Interval for OR**	
**Variables**	**B**	**Sh**	**Wald**	**Sd**	**p**	**Exp(B)**	**Lower**	**Upper**
Smoking Status (Ref: Non-smoker)	−0,811	0,173	21,931	1	0,001	0,444	0,317	0,624
Working in a Paid Job (Ref: Not Working)	−0,736	0,236	9,713	1	0,002	0,479	0,302	0,761
Health-Promoting and Protective Behaviors Scale (Continuous)	0,079	0,011	51,521	1	0,001	1,082	1,059	1,106
School Climate Scale (Continuous)	1,697	0,137	152,625	1	0,001	5,458	4,170	7,144
Type of Residence (Ref: Living with Family)			11,225	2	0,004			
Rented	−0,220	0,249	0,780	1	0,377	0,803	0,493	1,307
Dormitory	0,330	0,233	2,009	1	0,156	1,391	0,881	2,195

Nagelkerke R^2^ = 0,444; Omnibus Chi-square = 465,839; *p* < 0,001; Hosmer and Lemeshow = *p* > 0,05

## Dıscussıon

Based on the findings of this study, students’ perceptions of school climate were positively associated with both subjective and objective academic achievement. Students positive perceptions of the school climate were associated with higher levels of academic achievement, and this relationship was found to be statistically significant. Black et al. [11] reported that students with high rates of absenteeism tend to exhibit lower academic performance [11]. Manlove [43] reported that students with high school engagement exhibited lower rates of absenteeism and school dropout, which in turn were associated with higher academic achievement [43]. Daily et al. [19] observed that students with a positive perception of school climate exhibited higher academic achievement [19]. Gowing and Jackson [25] suggest that fostering a stronger sense of school belonging may better promote academic engagement [25]. Similarly, Jorgenson et al. [35] demonstrated that school engagement is a significant predictor of students’ academic achievement and their ability to maintain grades that effectively support university life [35]. Our findings, consistent with the existing literature, indicate that students with higher perceptions of school climate tend to report higher academic achievement.

In our study, a significant positive relationship was found between both subjective and objective academic achievement and promotive and protective health behaviors. The literature indicates that health-related behaviors are associated with academic performance, and this association is reflected in higher overall grade point averages, whereas health-risk behaviors such as substance use, sleep disturbances, and physical inactivity are also associated with lower academic achievement [39, 49, 61, 65]. Factors related to students health may be associated with psychological problems, which in turn may be associated with lower academic achievement [31]. Conditions such as anxiety, depression, or anxiety disorders may be associated with poorer health and may be linked to lower academic performance [36]. Halat et al. (2023) reported that high academic achievement is associated with multiple factors, including nutrition behavior, lifestyle, and mental health (Halat et al., 2023). Studies indicate that individuals who consume less alcohol tend to focus more on their academic work, and this pattern is associated with higher academic achievement. Similarly, academic performance tends to be lower in individuals who smoke compared to those who do not [64]. Pennanen et al. [48] reported that students with poor academic performance smoked six times more per week compared to students with high grade point averages. The researchers observed that increases in smoking behavior were associated with decreases in academic achievement [48]. Our study results are consistent with findings in the literature, indicating that health behaviors are associated with academic achievement.

In our study, individuals who regularly ate breakfast were found to have higher subjective and objective academic achievement. Doak et al. [21] demonstrated a relationship between poor dietary quality and higher body mass index, increased alcohol consumption, poorer sleep quality, higher stress levels, and smoking. All of these negative factors also pose a risk to academic achievement [21]. Barchitta et al. [9] reported that students who regularly consumed all main meals performed better compared to those who skipped at least one meal [9]. Reuter et al. [50] reported that healthy eating habits were associated with better academic performance, that breakfast consumption was associated with higher GPA, whereas fast food consumption was associated with lower GPA [50]. Our findings are consistent with studies in the literature.

In our study, individuals with regular sleep patterns exhibited higher subjective and objective academic achievement compared to those without regular sleep. Jalali et al. [33] reported that sleep duration, daytime sleepiness, and sleep quality were significantly associated with academic performance. Similarly, another study found that insufficient sleep was associated with lower academic performance in university students [27]. Research conducted on medical students indicated that nighttime sleep deprivation, late bedtimes, and daytime sleepiness were associated with poorer academic outcomes [8]. It has been reported that students cognitive performance is associated with sleep quality, and better sleep quality has been associated with higher academic achievement [47]. Other studies have also suggested that university students’ mental well-being, physical activity levels, interpersonal relationships, and overall daily quality of life have been associated with better sleep quality [1]. Consistent with the literature, this study observed that students with regular sleep habits reported higher academic achievement, more positive health behaviors, and more favorable perceptions of school climate.

In our study, individuals who engaged in regular exercise demonstrated higher subjective and objective academic achievement compared to those who did not exercise regularly. A study conducted among dental students reported that 60% of students were physically active while 40% were not, and the physically active students exhibited significantly higher academic performance than their inactive peers [5]. Similarly, Al-Drees et al. [3] found a positive relationship between physical activity levels and higher cumulative grade point averages among medical students [3]. Serel Arslan et al. [53] indicated that physical activity was associated with feeling healthier, coping better with stress, developing a sense of discipline, and maintaining more structured daily routines, and that increased physical activity is associated with higher academic achievement and reduced depression among students [53]. Hillman, Castelli, and Buck [29] reported that students with higher fitness levels demonstrated better cognitive performance, with aerobic fitness positively correlated with cognitive outcomes [29]. Our study findings, similar to those reported in the literature, suggest a positive relationship between physical activity and academic achievement.

In our study, individuals who had previously consulted a physician due to mental health issues had lower subjective and objective academic achievement. According to the literature, Anbari et al. [4] found a significant negative relationship between mental health scores and academic performance among medical, paramedical, nursing, and midwifery students [4]. Similarly, Maghalian et al. [41] reported a negative relationship between academic grade point average and social support, mental health scores, depression, anxiety, and cigarette and hookah consumption [41]. This finding aligns with evidence indicating that mental health challenges are associated with reduced academic performance.

In our study, when the relationship between subjective and objective academic achievement and gender was examined, no statistically significant relationship was observed. Engin-Demir [22] reported that women had higher average achievement scores than men [22]. Similarly, Novak et al. [46] observed that female students performed better than male students, and this difference was statistically significant [46]. Yıldırım et al. [67], in a study conducted with 1,376 university students, found that 43.4% of students with low academic achievement were women and 56.6% were men, indicating that women tended to have higher academic achievement than men [67]. Other studies have also shown that women achieve higher academic success than men [14, 54]. Although the literature generally reports higher academic performance among women, the lack of a significant gender difference in our study may be attributed to factors such as sample size, gender distribution, and the faculties in which the study was conducted.

In our study, it was observed that students who work in paid jobs have lower academic achievement compared to those who do not work. Hawkins et al. [28] reported that 35% of students with paid jobs indicated that their work significantly or greatly interfered with their studies. They observed that as the time spent working increased, the time allocated for studying decreased, and this pattern was associated with lower academic performance and grades [28]. Similarly, Richardson et al. [51] reported a negative relationship between work hours and academic achievement [51]. Our findings showed that students working in paid jobs had lower academic achievement. This is consistent with previous studies reporting that increased work hours are associated with reduced study time and lower academic performance.

In our study, when examining the relationship between students’ place of residence and academic achievement, it was observed that students living in rental accommodations had lower subjective and objective academic achievement compared to those living in dormitories or with their families, whereas students living with their families tended to report higher academic achievement. Büyüköztürk and Deryakulu [14] reported that students living in dormitories had lower academic performance compared to those living at home with friends [14]. Yıldırım et al. [67] noted that dormitory life provides advantages for academic success in terms of transportation, physical conditions, etc., and found that 34% of students with low academic achievement lived in dormitories while 66% lived at home [67]. The literature shows mixed results regarding the relationship between academic achievement and students type of residence. Differences across studies may reflect variations in sample characteristics and institutional contexts.

In our study, class level was not significantly associated with subjective academic achievement (SAAS). However, a statistically significant difference was observed between class level and objective academic achievement (GPA), with second-year students demonstrating higher GPA scores than third-year students. When class level was examined together with other variables in the logistic regression models, it did not remain a significant predictor and was not retained in the final models, indicating that class level did not independently explain variation in either subjective or objective academic achievement. Köse et al. [38] reported that 3rd-year students had lower academic achievement than 2nd-year students, with the difference between class levels being statistically significant, and found that university students in the 3rd year had lower academic performance [38]. Other studies in the literature also show that 2nd-year university students tend to have higher academic achievement (Arlı, 2013 [32],). As reported in previous studies, 2nd-year students tended to show higher academic achievement than 3rd-year students. The current study does not provide data to explain the causes of this difference, and further research is needed to clarify the underlying factors.

## Conclusion and recommendations

This study was conducted to investigate whether academic achievement among university students is influenced by factors such as school climate, health behaviors, demographic characteristics, living arrangements, and employment status. The findings, obtained from 1,155 students, indicate that academic success is a multidimensional concept shaped not only by exam results but also by cognitive, emotional, and environmental factors. In addition to the students’ cumulative grade point average, their perception of personal achievement is also an important indicator in assessing academic performance.

The study found a significant and strong relationship between students’ perceived academic achievement and their perceptions of school climate. A positive school climate is associated with greater attachment and motivation to school, which may be linked to higher academic achievement. Health-promoting and preventive behaviors were also found to support academic achievement. In particular, students who regularly eat breakfast, engage in physical exercise, and maintain a healthy sleep pattern exhibited higher levels of academic performance. Sleep quality was identified as a key factor, directly affecting attention, learning capacity, and cognitive performance, and therefore playing a critical role in academic success. Moreover, unhealthy behaviors such as smoking and alcohol consumption were found to negatively impact students’ academic performance. These habits have detrimental effects on both physical health and cognitive functions, thereby disrupting the learning process.

Within the scope of the study, it was also observed that students who work in paid employment may have lower academic achievement. Factors associated with working life, such as fatigue, stress, and limited time for studying, can negatively affect course performance. Consequently, numerous individual and environmental factors influence university students’ academic success. Academic achievement is shaped not only by students’ knowledge level but also by their lifestyle, health behaviors, social relationships, and connection with the university environment. Therefore, it is important for universities to implement initiatives that support healthy living habits, encouraging balanced nutrition, regular sleep, and physical activity. Enhancing students’ sense of belonging to the school and fostering a social and academic climate that promotes participation and engagement can positively impact achievement. Expanding mental health services, strengthening psychological support systems, and facilitating access to these services will contribute to students’ academic and personal development. Developing guidance mechanisms for students in need of academic support is crucial for improving success. Additionally, support measures such as scholarship opportunities, orientation programs, and tailored interventions for working students or those coming from other cities should be widely implemented. Policies aimed at enhancing academic performance should not be limited to curriculum content alone; they should adopt a holistic approach encompassing students’ adaptation to school, the quality of educational and communication environments, school engagement, living conditions, psychological well-being, social support needs, and healthy lifestyle behaviors. When the motivation theories in the literature are examined, it is seen that our findings are compatible with well-established theoretical frameworks. According to Self-Determination Theory [52], a positive school climate and healthy behavioral patterns may support students’ basic psychological needs—autonomy, competence, and relatedness—which may contribute to the development of more intrinsic and sustainable motivation toward learning. In addition, when evaluated from the perspective of Expectancy–Value Theory [63], supportive educational environments and healthy lifestyle patterns may strengthen students’ expectations of academic success and their perceptions of the value of academic effort. These theoretical approaches assist in evaluating the findings obtained in our study within a broader framework.

### Limitations of the study

During the course of the study, some students were unable to fully respond to the items on the scales due to language barriers. This resulted in incomplete or inaccurate data, which could not be included in the study. Additionally, students enrolled in mathematics-related programs had lower class attendance rates, making it difficult to reach the planned sample size for these fields. Due to the irregular attendance of many students in these faculties and the limited number of students accessible for direct contact within the scope of the study, proportionally fewer students could be reached in faculties with low class attendance. Consequently, the study was conducted only with students who regularly attended classes, and data from absentee students could not be obtained. Therefore, information regarding the academic performance of students who did not attend classes could not be determined. In addition, the use of convenience sampling and the uneven representation of faculties, particularly the underrepresentation of mathematical sciences, may have introduced selection bias. Because academic achievement, school climate perceptions, and health behaviors can differ across faculties, such imbalances may affect internal validity and regression estimates. This imbalance may lead to biased coefficient estimates or over-/under-estimation of associations. Future studies are recommended to use probability-based or weighted sampling methods to reduce sampling bias.

The SAAS was adapted and psychometrically assessed within our sample (Cronbach’s α = 0.837). However, these analyses are sample-specific; broader, multicenter validation studies are required to confirm the scale’s generalisability to other university populations. Additionally, the study relied on students’ self-reported responses, which may be influenced by factors such as social desirability bias, recall bias, or misrepresentation of oneself. Furthermore, due to the lack of access to updated student lists for each department, probability sampling methods could not be applied. This limitation may have prevented the sample from fully representing the population, thereby restricting the generalizability of the findings.

In the study, participants breakfast habits were assessed only as “yes” or “no,” and detailed aspects such as the frequency of breakfast consumption, meal composition, or nutritional quality could not be examined.

## Supplementary Information

Supplementary Material 1.

Supplementary Material 2.

Supplementary Material 3.

## Data Availability

The dataset is available from the corresponding author upon reasonable request.

## Abbreviations

SAAS: Subjective Academic Achievement Scale
GPA: Grade Point Average
