# Supplementary material for: The relationship between academic achievement, health behaviors, and school climate among university students
Source: BMC Public Health. 2026 Jan 20;26:1112. doi: 10.1186/s12889-026-26324-5 (PMC13059555; doi:10.1186/s12889-026-26324-5)
Supplement: Supplementary file 1 — Supplementary Material 1. [file 12889_2026_26324_MOESM1_ESM.docx]

**Section A — Socio-demographic information**

1. Participant ID: _______
2. Age (years): _____
3. Gender: 1=Female 2=Male 3=Other/Prefer not to say
4. Faculty/Department: ___________________
5. Year of study: 2nd year / 3rd year
6. Current GPA: _____ (e.g., 2.60)
7. Accommodation type: 1=Dormitory 2=With family 3=Rented
8. Employment in income-generating job: 1=Yes 2=No
9. Have you ever consulted a physician for mental health reasons? 1=Yes 2=No
10. Do you smoke? 1=Yes 2=No
11. Do you consume alcohol? 1=Yes 2=No
12. Do you eat breakfast regularly? 1=Yes 2=No
13. Do you exercise regularly? 1=Yes 2=No
14. How would you rate your general health? 1=Good 2=Moderate 3=Poor
15. How would you describe your sleep pattern? 1=Regular (healthy sleep) 2=Irregular

**Section B — SAAS** (see Stadler et al., 2021)
**Section C — School Climate Scale** (see Terzi, 2015)
**Section D — Health-Promoting and Protective Behaviors Scale** (see Bostan et al., 2016)
