# Supplementary material for: The relationship between academic achievement, health behaviors, and school climate among university students
Source: BMC Public Health. 2026 Jan 20;26:1112. doi: 10.1186/s12889-026-26324-5 (PMC13059555; doi:10.1186/s12889-026-26324-5)
Supplement: Supplementary file 2 — Supplementary Material 2. [file 12889_2026_26324_MOESM2_ESM.docx]

**SAAS PSYCHOMETRİC DETAİLS**

Recommended contents (please fill with your exact results):

- Sample size (n = **100**)
- KMO statistic = **0,801** ; Bartlett’s test p < 0.001
- EFA method: Principal Components Analysis; rotation: Varimax (rotated solution)
- Number of extracted factors = 1; total explained variance = 58,65%
- **Table**: Cronbach’s Alpha Values of the Subjective Academic Achievement Scale

| **Item** | **Factor 1 Loading** | **Item-Total Correlation** | **Cronbach’s Alpha if Item Deleted** |
| --- | --- | --- | --- |
| Item 1 | 0,772 | 0,623 | 0,783 |
| Item 2 | 0,708 | 0,550 | 0,805 |
| Item 3 | 0,791 | 0,641 | 0,778 |
| Item 4 | 0,771 | 0,628 | 0,782 |
| Item 5 | 0,785 | 0,640 | 0,782 |

- Cronbach’s α for each factor and for total scale (α = **0,821**)
- If CFA performed: Not performed

**Title:** SAAS Psychometric Report — Example results (n = 100)

**Psychometric evaluation of the Subjective Academic Achievement Scale (SAAS) — Example results (n = 100)**

The Subjective Academic Achievement Scale (SAAS; 5 items) was evaluated in the full sample (n = 100). Item distributions showed no extreme floor or ceiling effects. Sampling adequacy was acceptable: KMO = 0.801 and Bartlett’s test of sphericity χ²(10) = 165.494, p < 0.001. Parallel analysis and the scree plot supported a single-factor solution. An exploratory factor analysis (principal component analysis, one factor extracted) returned one factor explaining 58.65% of the total variance. Factor loadings ranged from 0.708 to 0.791, and communalities ranged from 0.501 to 0.626.

Internal consistency was satisfactory: Cronbach’s α = 0.821 for the full scale. Item-total correlations ranged 0.550–0.641 and “α if item deleted” values varied between 0.778 and 0.805, indicating no item materially degraded the total scale reliability. Based on these results, the SAAS performs adequately in this sample as a unidimensional measure of subjective academic achievement. Detailed item statistics and factor loadings are provided in Supplementary File S2.

**Table 1 (illustrative) — Item statistics and EFA loadings (n = 100)**

| Item | mean | sd | Item-total r | Factor loading | Communality | α if item deleted |
| --- | --- | --- | --- | --- | --- | --- |
| 1 | 3,070 | 1,103 | 0,623 | 0,772 | 0,596 | 0,783 |
| 2 | 3,190 | 1,098 | 0,550 | 0,708 | 0,501 | 0,805 |
| 3 | 3,150 | 1,192 | 0,641 | 0,791 | 0,626 | 0,778 |
| 4 | 3,100 | 1,133 | 0,628 | 0,771 | 0,594 | 0,782 |
| 5 | 2,920 | 0,950 | 0,640 | 0,758 | 0,616 | 0,782 |

Scale-level: Cronbach’s α = 0.821; total variance explained by factor 1 = 58.65%; KMO = 0.801; Bartlett’s χ²(10) = 165.494, p < 0.001.
