# Supplementary material for: The relationship between academic achievement, health behaviors, and school climate among university students
Source: BMC Public Health. 2026 Jan 20;26:1112. doi: 10.1186/s12889-026-26324-5 (PMC13059555; doi:10.1186/s12889-026-26324-5)
Supplement: Supplementary file 3 — Supplementary Material 3. [file 12889_2026_26324_MOESM3_ESM.docx]

Editor: Third, the Results section is overly descriptive and redundant, with excessive tables that repeat information; these should be streamlined, prioritizing key findings in the main text and moving supplementary details to an appendix.

Response: These tables were moved from the main Results section upon reviewer request to reduce redundancy

Table 1 presents descriptive statistics for the continuous variables evaluated in the study. In addition to the participants’ mean age, body mass index (BMI), GPA, and scores on the Subjective Academic Achievement Scale (SAAS), the table includes mean and median values (min–max) for the School Climate Scale and the Health-Promoting and Protective Behaviors Scale.

**Table 1.** Means and Distributions of University Students’ Academic Achievement, School Climate, Health-Promoting and Protective Behaviors, and Socio-Demographic Characteristics

|  | **Mean ± ss** | **Median (Min–Max)** |
| --- | --- | --- |
| **GPA** | 2,59 ± 0,42 | 2,60 (1,40-3,73) |
| **Subjective Academic Achievement Scale** | 15,46 ± 3,86 | 16,00 (5,00-25,00) |
| **School Climate Scale** | 3,19 ± 0,69 | 3,23 (1,00-5,00) |
| **Health-Promoting and Protective Behaviors Scale** | 77,10 ± 7,96 | 77,00 (49,00-100,00) |
| **Age (years)** | 21,80 ± 2,88 | 21 (18-61) |
| **Body Mass Index** | 23,24 ± 3,81 | 22,84 (15,32-42,06) |

Table 2 presents the factors associated with subjective academic achievement. Participants who smoked (χ² = 37.387, p < 0.001), consumed alcohol (χ² = 12.307, p < 0.001), did not eat breakfast regularly (χ² = 20.945, p < 0.001), had irregular sleep patterns (χ² = 36.430, p < 0.001), did not exercise regularly (χ² = 7.416, p = 0.006), had a history of consulting a physician for mental health issues (χ² = 25.032, p < 0.001), reported poor or moderate general health status (χ² = 73.863, p < 0.001), were employed in income-generating jobs (χ² = 7.995, p = 0.005), lived in rented accommodation (χ² = 14.987, p < 0.001), perceived a negative school climate (χ² = 297.618, p < 0.001), and had low levels of health-promoting and protective behaviors (χ² = 115.091, p < 0.001) reported lower subjective academic achievement scores. No significant differences were observed in relation to gender (χ² = 2.269, p = 0.132), class level (χ² = 2.301, p = 0.129), or department type (χ² = 0.161, p = 0.923).

**Table 2.** Subjective Academic Success and Associated Factors

| **Variable** | | **Subjective Academic Achievement** | |  |  |
| --- | --- | --- | --- | --- | --- |
|  |  | **≤16** | **>16** | **χ²** | **p** |
| **Gender** | Female | 358 (%60,6) | 317(%56,2) | 2,269 | 0,132 |
|  | Male | 233 (%39,4) | 247(%43,8) |  |  |
| **Smoking** | Yes | 246(%72,1) | 95(%27,9) | 37,387 | 0,001 |
|  | No | 429(%52,7) | 385(%47,3) |  |  |
| **Alcohol Use** | Yes | 141(%69,5) | 62(%30,5) | 12,307 | 0,001 |
|  | No | 534(%56,1) | 418(%43,9) |  |  |
| **Regular Breakfast** | Yes | 346(%52,7) | 311(%47,3) | 20,945 | 0,001 |
|  | No | 329(%66,1) | 169(%33,9) |  |  |
| **Sleep Pattern** | Regular | 249(%48,6) | 263(%51,4) | 36,43 | 0,001 |
|  | Irregular | 426(%66,3) | 217(%33,7) |  |  |
| **Regular Exercise** | Yes | 349(%54,9) | 287(%45,1) | 7,416 | 0,006 |
|  | No | 326(%62,8) | 193(%37,2) |  |  |
| **History of Mental Health Consultation** | Yes | 146 (%74,5) | 50 (%25,5) | 25,032 | 0,001 |
|  | No | 529 (%55,2) | 430 (%44,8) |  |  |
| **General Health Status** | Good | 297^a^ (%47,3) | 330^a^ (%52,7) | 73,863 | 0,001 |
|  | Moderate | 312^b^ (%69,6) | 136^b^ (%30,4 ) |  |  |
|  | Poor | 66^c^ (%82,5) | 14^c^ (%17,5) |  |  |
| **Employment** | Employed | 113(%68,5) | 52(%31,5) | 7,995 | 0,005 |
|  | Unemployed | 562(%56,8) | 428(%43,2) | 14,987 | 0,001 |
| **Accommodation Type** | Dormitory | 234^a^ (%65,2) | 125^a^ (%34,8) |  |  |
|  | With Family | 374^b^ (%57,3) | 279^b^ (%42,7) |  |  |
|  | Rented | 67^c^ (%46,9) | 76^c^ (%53,1) |  |  |
| **Class Level** | 2nd Year | 321(%56,2) | 250(%43,8) | 2,301 | 0,129 |
|  | 3nd Year | 354(%60,6) | 230(%39,4) |  |  |
| **Department Type** | Social | 213^a^ (%57,7) | 156^a^ (%42,3) | 0,161 | 0,923 |
|  | Mathematical | 236^a^ (%59,1) | 163^a^ (%40,9) |  |  |
|  | Health | 226^a^ (%58,4) | 161^a^ (%41,6) |  |  |
| **School Climate** | Poor | 216^a^ (%90,8) | 22^a^ (%9,2) | 297,618 | 0,001 |
|  | Moderate | 313^b^ (%72,5) | 119^b^ (%27,5) |  |  |
|  | Good | 146^c^ (%30,1) | 339^c^ (%69,9) |  |  |
| **Health-Promoting and Protective Behaviors Scale** | ≤77 | 441 (%73,4) | 160 (%26,6) | 115,091 | 0,001 |
|  | >77 | 234 (%42,2) | 320 (%57,8) |  |  |

Table 3 shows the results of the analysis examining the factors associated with the grade point average. Participants who smoked (χ² = 79.233, p < 0.001), consumed alcohol (χ² = 23.615, p < 0.001), did not eat breakfast regularly (χ² = 25.012, p < 0.001), had irregular sleep patterns (χ² = 25.649, p < 0.001), did not exercise regularly (χ² = 10.736, p < 0.001), had a history of consulting a physician for mental health issues (χ² = 15.597, p < 0.001), reported moderate or poor general health status (χ² = 78.924, p < 0.001), were employed in income-generating jobs (χ² = 28.891, p < 0.001), lived in rented accommodation (χ² = 29.528, p < 0.001), were third-year students (χ² = 5.854, p = 0.016), studied in social or mathematical departments (χ² = 45.838, p < 0.001), perceived a negative school climate (χ² = 296.821, p < 0.001), and had low scores on the Health-Promoting and Protective Behaviors Scale (χ² = 151.944, p < 0.001) demonstrated lower objective academic achievement (GPA). No significant differences were observed in relation to gender (χ² = 3.704, p = 0.054).

**Table 3.** GPA and Associated Factors

| **Variable** | | **GPA** | | |  |
| --- | --- | --- | --- | --- | --- |
|  |  | **≤2,60** | **>2,60** | **χ²** | **p** |
| **Gender** | Female | 303(%51,3) | 288(%48,7) | 3,704 | 0,054 |
|  | Male | 321(%56,9) | 243(%43,1) |  |  |
| **Smoking** | Yes | 253(%74,2) | 88(%25,8) | 79,233 | 0,001 |
|  | No | 371(%45,6) | 443(%54,4) |  |  |
| **Alcohol Use** | Yes | 141(%69,5) | 62(%30,5) | 23,615 | 0,001 |
|  | No | 483(%50,7) | 469(%49,3) |  |  |
| **Regular Breakfast** | Yes | 313(%47,6) | 344(%52,4) | 25,012 | 0,001 |
|  | No | 311(%62,4) | 187(%37,6) |  |  |
| **Sleep Pattern** | Regular | 234(%45,7) | 278(%54,3) | 25,649 | 0,001 |
|  | Irregular | 390(%60,7) | 253(%39,3) |  |  |
| **Regular Exercise** | Yes | 316(%49,7) | 320(%50,3) | 10,736 | 0,001 |
|  | No | 308(%59,3) | 211(%40,7) |  |  |
| **History of Mental Health Consultation** | Yes | 131 (%66,8) | 493 (%51,4) | 15,597 | 0,001 |
|  | No | 65 (%33,2) | 466 (%48,6) |  |  |
| **General Health Status** | Good | 268^a^ (%42,7) | 359^a^ (%57,3) | 78,924 | 0,001 |
|  | Moderate | 290^b^ (%64,7) | 158^b^ (%35,3) |  |  |
|  | Poor | 66^c^ (%82,5) | 14^c^ (%17,5) |  |  |
| **Employment** | Employed | 121 (%73,3) | 44 (%26,7) | 28,891 | 0,001 |
|  | Unemployed | 503 (%50,8) | 487 (%49,2) |  |  |
| **Accommodation Type** | Dormitory | 236^a^ (%65,7) | 123^a^ (%34,3) | 29,528 | 0,001 |
|  | With Family | 323^b^ (%49,5) | 330^b^ (%50,5) |  |  |
|  | Rented | 65^b^ (%45,5) | 78^b^ (%54,5) |  |  |
| **Class Level** | 2nd Year | 288(%50,4) | 283(%49,6) | 5,854 | 0,016 |
|  | 3nd Year | 336(%57,5) | 248(%42,5) |  |  |
| **Department Type** | Social | 201^a^ (%54,5) | 168^a^ (%45,5) | 45,838 | 0,001 |
|  | Mathematical | 262^b^ (%65,7) | 137^b^ (%34,3) |  |  |
|  | Health | 161^c^ (%41,6) | 226^c^ (%58,4) |  |  |
| **School Climate** | Poor | 210^a^ (%88,2) | 28^a^ (%11,8) | 296,821 | 0,001 |
|  | Moderate | 289^b^ (%66,9) | 143^b^ (%33,1) |  |  |
|  | Good | 125^c^ (%25,8) | 360^c^ (%74,2) |  |  |
| **Health-Promoting and Protective Behaviors Scale** | ≤77 | 429 (%71,4) | 172 (%28,6) | 151,944 | 0,001 |
|  | >77 | 195 (%35,2) | 359 (%64,8) |  |  |
